# Non-Hermitian indirect exchange interaction in a topological insulator coupled to a ferromagnetic metal

**DOI:** 10.1038/s41598-021-01591-x

**Published:** 2021-11-12

**Authors:** Mir Vahid Hosseini, Mehdi Askari

**Affiliations:** 1 Department of Physics, Faculty of Science, University of Zanjan, Zanjan, 45371-38791 Iran; 2 Department of Physics, Faculty of Science, Salman Farsi University of Kazerun, Kazerun, Iran

**Keywords:** Topological insulators, Magnetic properties and materials, Condensed-matter physics, Spintronics

## Abstract

We theoretically demonstrate non-Hermitian indirect interaction between two magnetic impurities placed at the interface between a 3D topological insulator and a ferromagnetic metal. The coupling of topological insulator and the ferromagnet introduces not only Zeeman exchange field on the surface states but also broadening to transfer the charge and spin between the surface states of the topological insulator and the metallic states of the ferromagnet. While the former provides bandgap at the charge neutrality point, the latter causes non-Hermiticity. Using the Green’s function method, we calculate the range functions of magnetic impurity interactions. We show that the charge decay rate provides a coupling between evanescent modes near the bandgap and traveling modes near the band edge. However, the spin decay rate induces a stronger coupling than the charge decay rate so that higher energy traveling modes can be coupled to lower energy evanescent ones. This results in a non-monotonic behavior of the range functions in terms of distance and decay rates in the subgap regime. In the over gap regime, depending on the type of decay rate and on the distance, the amplitude of spatial oscillations would be damped or promoted.

## Introduction

Non-Hermitian physics has provided an environment in which more realistic physical systems can be studied^1^. Many realistic systems are not isolated systems, but rather are open ones^2–5^ meaning that the probability for them is not conserved owing to exchange of energy, particle, and information with external reservoirs. Recently, non-Hermitian systems have been attracted a lot of attention^6^. Several unconventional physical properties have been identified by including non-hermiticity in both classical^7–9^ and quantum^10–14^ regimes which are absent in their Hermitian counterparts. Generally, open quantum systems can be described by non-Hermitian Hamiltonians which may have complex eigenvalues^15^. So, the development of non-Hermiticity to topologically nontrivial band structures^16^, such as topological insulators^17^, topological superconductors^18^, and etc., is indeed an interesting issue, particularly in condensed matter physics.

Topological insulators^17^ have received a lot of attention due to their amazing nontrivial electronic states protected by time-reversal symmetry. In these materials, while bulk states are gapped, surface states are gapless and chiral because of the nontrivial topology originating from the strong spin-orbit interaction^19^. Due to chirality, the momentum and spin of carriers are locked together. Also, the chiral surface states in topological insulators resemble relativistic particles in high energy physics so that particle-like and hole-like states touch each other at Dirac points. These features result in magnetoelectric polarizability^20^, magnetic monopole induction^21^, and magnetic-impurity-induced local gap^22,23^ in topological insulators. Although the surface states of topological insulator are topologically protected, but there are some approaches that make the engineering of these states possible. For instance, an energy gap in the topological insulator surface states can be generated by breaking the time-reversal symmetry through proximity coupling to a ferromagnetic metal^24^ and magnetic doping^25–28^. In the former case, the escaping of charge carriers from the topological insulator to the ferromagnetic metal makes the system non-Hermitian. This can result in the appearance of a non-symmetry-protected non-Hermitian Weyl phase characterized by bulk Fermi arc which can be manipulated by magnetization direction^29^. While, in the latter case, the gap is not robust and can be filled by potential scattering^30^. Recently, it has been shown that the topological insulator surface states can sustain the magnetic ordering while the bulk states would be spoiled in Cr-doped Bi$_2$Se$_3$ thin film^31^, indicating that impurity interactions depend on whether magnetic impurities are on the surface or in the bulk of system.

Indirect exchange interaction between magnetic impurities mediated by carriers of host material, known as Ruderman–Kittel–Kasuya–Yosida (RKKY) interaction^32–34^ has been investigated in systems having definite chirality, such as graphene^35–44^, 3D topological insulators^45–47^, topological crystalline insulators^48,49^, and Weyl quasi-particles^50,51^. The effects of superconducting correlations^52^, temperature, and Zeeman field^53^ on the RKKY interaction have also been explored in topological insulators. However, the study is still lacking as far as the signature of non-hermiticity in magnetic indirect interaction is concerned. Recently, nonreciprocal exchange coupling mediated by magnons, i.e., spin waves, has been studied between two separated ferromagnetic nanowires in a lateral structure^54^. So, it is interesting to know that how two magnetic adatoms interact through Dirac fermions, i.e., electronic waves, in a non-Hermitian topological insulator.

In the present work, we develop the theory of indirect exchange interaction to a non-Hermitian case in such a way that magnetic adatoms are placed at the interface of a 3D topological insulator and a ferromagnetic metal. The coupling between the topological insulator surface states and the ferromagnetic metal introduces non-Hermitian terms, i.e., charge and spin decay rates, being responsible for transferring charge and spin between the two subsystems. Employing Green’s function method, we find that the resulting interaction is comprised of in-plane spin-frustrated interactions being along and perpendicular to the line connecting the two magnetic impurities, Dzyaloshinsky-Moriya interaction, and out-of-plane Ising interaction. We also show that, in the presence of charge decay rate, the traveling modes near the band edge can couple to the evanescent modes near the bandgap edge such that the amplitude of range functions increases and then decreases as a function of charge decay rate at intermediate distances. While, in the presence of spin decay rate, the coupling between both types of modes is so strong that the modes far away from the band edge or bandgap can be coupled together so that the amplitude of range functions oscillates explicitly as a function of spin decay rate even at intermediate distances. Correspondingly, the spatial dependence of the range functions would be affected due to decay rates: In the subgap regime, in addition to the exponentially decaying behavior of the envelope function of range functions, there are sign changes versus distance for the finite value of the decay rates. In the over gap regime, the charge decay rate damps the usual spatially oscillatory behavior of the range functions, whereas the spin decay rate damps the oscillations at small distances but promotes them at large distances. Furthermore, we analytically obtain asymptotic expressions for the range functions in the short-range and long-range limits.

The paper is organized as follows. In “Model and theory”, we introduce the model and extract the types of magnetic impurity interactions mediated by interface electrons of ferromagnetic metal/tpological insulator heterostructure in the presence of charge and spin decay rates. In “Numerical results”, we numerically evaluate the obtained range functions and study their dependence on various related parameters. Analytical expressions of the range functions are derived in “Analytical results”. Finally, we summarize and conclude in “Summary”.

**Figure 1:**
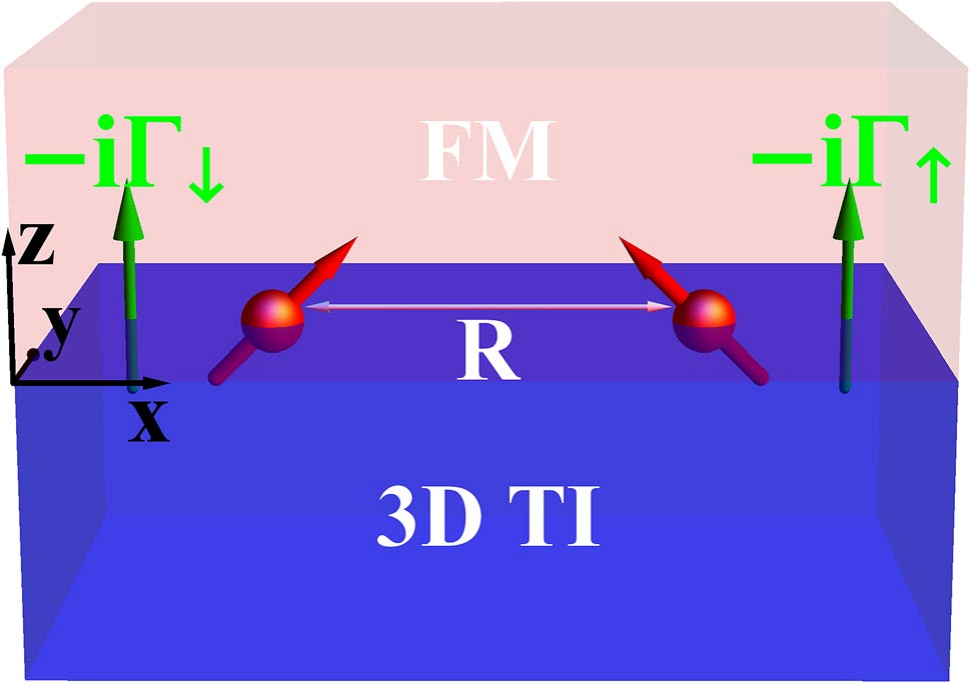
(Color online) Heterostructure consisting of a 3D topological insulator coupled to a ferromagnetic metal. There are two magnetic impurities at the interface that are separated by a distance *R*. The system is also influenced by the Zeeman exchange field and spin dependent decay rates $\Gamma _{\uparrow }$ and $\Gamma _{\downarrow }$.

## Model and theory

We consider a ferromagnetic metal on top of a 3D topological insulator, as shown in Fig. 1. The interface of junction containing magnetic impurities is placed on the *xy*-plane. To describe the surface states of topological insulator in the presence of a proximity-coupled ferromagnet, we use the following low-energy effective non-Hermitian Hamiltonian^24,55^

1 $$\begin{aligned} H_0({\mathbf {k}}) =-i\Gamma _0\sigma _0+ v_F (k_y\sigma _x - k_x\sigma _y) - (M+i\Gamma _z)\sigma _z, \end{aligned}$$

where *M* is the proximity-induced exchange field breaking time-reversal symmetry due to the metallic ferromagnet. $v_F$ is the Fermi velocity. Also, $\sigma _{x,y,z}$ and $\sigma _{0}$ are the Pauli matrices and identity matrix, respectively, acting on the spin space. $\Gamma _0=(\Gamma _{\uparrow }+\Gamma _{\downarrow })/2$ and $\Gamma _z=(\Gamma _{\uparrow }-\Gamma _{\downarrow })/2$, providing the non-Hermitian terms in Eq. (1), are the charge and spin broadenings, respectively, with the spin dependent decay rates $\Gamma _{\uparrow (\downarrow )}$. In fact, the non-Hermitian terms originate from the coupling of the ferromagnet to the topological insulator surface states through surface self-energies to include the effect of the semi-infinite ferromagnetic metal^24^. In order to take into account the metallic bands of ferromagnet considerably, we have assumed that the metallic bands are centered at Dirac point, i.e., E=0. Also, low-energy approximation has been applied on the metallic bands of ferromagnet similar to those of the topological insulator. This causes that the non-Hermitian terms take constant values^24^. For convenience, throughout the paper, we set $\hbar$ = 1 and, without loss of generality, we assume $\Gamma _0,\Gamma _z\ge 0$. Diagonalizing Eq. (1), yields spectra as

2 $$\begin{aligned} \varepsilon (k)= & {} l\frac{\sqrt{\zeta + \sqrt{\zeta ^2+(2M\Gamma _z)^2}}}{\sqrt{2}}+i\left( l\frac{\sqrt{-\zeta + \sqrt{\zeta ^2+(2M\Gamma _z)^2}}}{\sqrt{2}}-\Gamma _0\right) , \end{aligned}$$

where $l=-(+)$ is the band index and $\zeta =(v_Fk)^2+M^2-\Gamma _z^2$ with $k=\sqrt{k^2_x+k^2_y}$.

**Figure 2:**
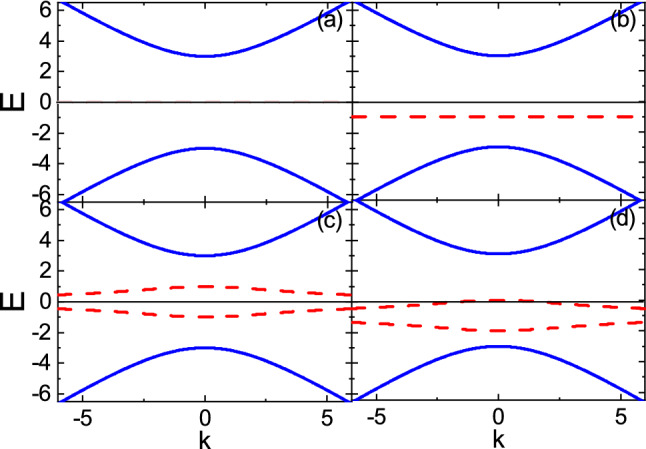
(Color online) Dispersion relation of the system surface states for **(a)** $\Gamma _0=0$ and $\Gamma _z=0$, **(b)** $\Gamma _0=1$ and $\Gamma _z=0$ **(c)** $\Gamma _0=0$ and $\Gamma _z=1$, and **(d)** $\Gamma _0=1$ and $\Gamma _z=1$, with $M=3$. The solid and dashed lines indicate the real and imaginary parts of spectra, respectively. The bandgap width is 2*M*.

Obviously, the spectra, Eq. (2), have real and imaginary parts. In Fig. 2, the real and imaginary parts of spectra as a function of *k* are represented by solid and dashed lines, respectively. *M* lifts the Dirac point degeneracy being responsible for opening the bandgap of width 2*M*. For $\Gamma _0=\Gamma _z=0$, the imaginary part disappears and the real part reduces to the usual gapped Dirac spectrum (see Fig. 2a). $\Gamma _0$ indicates the energy level of the imaginary part (see Fig. 2b). Whereas $\Gamma _z$ splits the imaginary energy states by breaking their band degeneracy (see Fig. 2c,d).

We consider a pair of magnetic impurities, ${\mathbf {S}}_1$ and ${\mathbf {S}}_2$ located at positions ${\mathbf {R}}_1$ and ${\mathbf {R}}_2$, respectively, at the interface. The interaction between the impurity magnetic moments and the itinerant fermions is modeled as a local potential at the impurity sites

3 $$\begin{aligned} H_{\text {int}} = \sum _{i=1,2} \sum _{j=x,y,z} J_j {\mathbf {S}}^j_i \sigma ^j \delta ({\mathbf {r}} - {\mathbf {R}}_i), \end{aligned}$$

where $J_j$ is the coupling strength between the impurities and the surface state fermions. Due to the lack of reflection symmetry with respect to the plane of interface, we assume the interaction to be anisotropic such that $J_x=J_y\ne J_z$. Employing perturbation theory and treating $H_{\text {int}}$ as a perturbation to $H_0$, up to second order of perturbation and at zero temperature, the indirect exchange interaction between the two magnetic impurities mediated by host fermions can be expressed by^32–34,56^

4 $$\begin{aligned} \begin{aligned} H_{\text {RKKY}} = -\frac{1}{\pi }\sum _{j,k} Im\int _{-\infty }^{E_f} d\varepsilon Tr [J_j{\mathbf {S}}^j_1\sigma ^j G({\mathbf {R}},\varepsilon ^+)J_k{\mathbf {S}}^k_2\sigma ^k G(-{\mathbf {R}},\varepsilon ^+)], \end{aligned} \end{aligned}$$

where $\varepsilon ^+ = \varepsilon +i 0^+$, *Tr* represents the trace over the spin degree of freedom, *Im* means imaginary part, $E_f$ is the Fermi energy measured from charge neutrality point, i.e., $E=0$, and ${\mathbf {R}} = {\mathbf {R}}_2 - {\mathbf {R}}_1$. The Green’s function in the energy-coordinate representation $G({\mathbf {R}},\varepsilon ^+)$ can be obtained from the Fourier transform,

5 $$\begin{aligned} G({\mathbf {R}}, \varepsilon ^+)= & {} \int \frac{d^2k}{(2\pi )^2} \ e^{i \mathbf {{\mathbf {k}}} \cdot {\mathbf {R}}} G({\mathbf {k}}, \varepsilon ^+), \end{aligned}$$

with

6 $$\begin{aligned} G({\mathbf {k}}, \varepsilon ^+) = [\varepsilon ^+ - H_0({\mathbf {k}})]^{-1}, \end{aligned}$$

where $G({\mathbf {k}}, \varepsilon ^+)$ is the momentum space Green’s function.

Using Eq. (1) and plugging Eq. (6) into Eq. (5), we carry out Fourier transform and obtain the real-space Green’s function as

7 $$\begin{aligned} G(\pm {\mathbf {R}}, \varepsilon ^+)= & {} G_0\sigma _0\pm G_I(\varvec{\sigma }\times {\hat{n}})\cdot {\hat{z}}+G_z\sigma _z, \end{aligned}$$

where

8 $$\begin{aligned} G_0= & {} -\frac{i}{4 v_F^2}(\varepsilon ^++i\Gamma _0)H^{(1)}_0\left( \frac{\Lambda R}{v_F}\right) , \end{aligned}$$

9 $$\begin{aligned} G_I= & {} -\frac{\Lambda }{4 v_F^2}H^{(1)}_1\left( \frac{\Lambda R}{v_F}\right) ,\end{aligned}$$

10 $$\begin{aligned} G_z= & {} \frac{i}{4 v_F^2}(M+i\Gamma _z)H^{(1)}_0\left( \frac{\Lambda R}{v_F}\right) , \end{aligned}$$

and

11 $$\begin{aligned} \Lambda =sgn(\varepsilon ^+-\frac{M\Gamma _z}{\Gamma _0}) \sqrt{(\varepsilon ^++i\Gamma _0)^2-(M+i\Gamma _z)^2}, \end{aligned}$$

with ${\hat{n}}={\mathbf {R}}/R$ a unit vector on the xy-plane along the line connecting the two localized impurities and $H^{(1)}_n(x)$ the first kind Hankel function of order *n*. It is worthwhile mentioning that the form of $\Lambda$ implies the wave vector as well as Fermi momentum can be a complex quantity not only in the subgap regime but also in the over gap regime, unlike other gapped systems. Note that the z component of Green’s function, i.e., $G_z$, originates from the exchange field *M* and $\Gamma _z$ explicitly. Substituting Eq. (7) into Eq. (4), the resulting indirect exchange interaction can be written as

12 $$\begin{aligned} H_{\text {RKKY}} =F_1({\mathbf {S}}_1 \cdot {\hat{n}}) ({\mathbf {S}}_2 \cdot {\hat{n}}) + F_2({\mathbf {S}}_1 \cdot {\hat{\rho }}) ({\mathbf {S}}_2 \cdot {\hat{\rho }}) + F_3 [({\mathbf {S}}_1 \times {\mathbf {S}}_2)\times {\hat{n}}]\cdot {\hat{z}} + F_4{\mathbf {S}}^z_1 {\mathbf {S}}^z_2, \end{aligned}$$

where ${\hat{\rho }}= {\hat{z}} \times {\hat{n}}$ is an in-plane unit vector perpendicular to ${\hat{n}}$. Here, the range functions are defined by

13 $$\begin{aligned} F_1(R,E_f)= & {} -\frac{2J^2_x}{\pi } Im \int _{-\infty }^{E_f} (G^2_0+G^2_I-G^2_z)d\varepsilon , \end{aligned}$$

14 $$\begin{aligned} F_2(R,E_f)= & {} -\frac{2J^2_x}{\pi } Im \int _{-\infty }^{E_f} (G^2_0-G^2_I-G^2_z)d\varepsilon ,\end{aligned}$$

15 $$\begin{aligned} F_3(R,E_f)= & {} \frac{4J_xJ_z}{\pi } Im \int _{-\infty }^{E_f} iG_0G_Id\varepsilon ,\end{aligned}$$

16 $$\begin{aligned} F_4(R,E_f)= & {} -\frac{2J^2_z}{\pi } Im \int _{-\infty }^{E_f} (G^2_0+G^2_I+G^2_z)d\varepsilon . \end{aligned}$$

**Figure 3:**
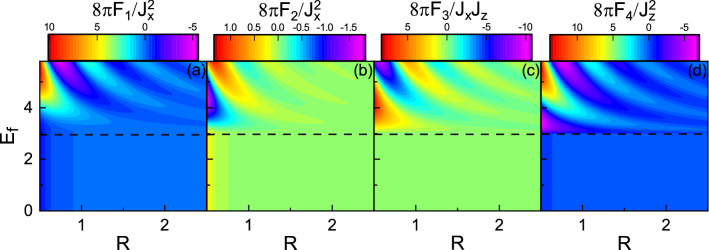
(Color online) **(a)** $F_1$, **(b)** $F_2$, **(c)** $F_3$, and **(d)** $F_4$ as functions of *R* and $E_f$ with $M=3$, $\Gamma _0=0$, and $\Gamma _z=0$. The horizontal dashed line indicates $E_f=M$.

**Figure 4:**
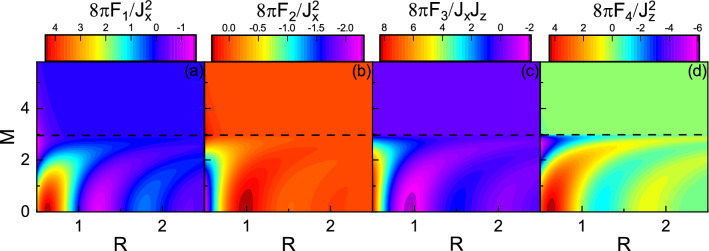
(Color online) **(a)** $F_1$, **(b)** $F_2$, **(c)** $F_3$, and **(d)** $F_4$ as functions of *R* and *M* with $E_f=3$, $\Gamma _0=0$, and $\Gamma _z=0$. The horizontal dashed line indicates $E_f=M$.

On the right-hand side of Eq. (12), the first and the second terms, describing the spin-frustrated interaction, cause an in-plane collinear magnetic ordering with spin orientation along and perpendicular to the line connecting the two impurities, respectively. The third term, referred to as Dzyaloshinsky–Moriya interaction, favors an in-plane non-collinear magnetic spin ordering. The last term, known as Ising interaction, depending on its sign imposes out-of-plane (anti) parallel alignment of the impurity spins. Remarkably, from Eqs. (13)–(16), one finds that $F_3$ can be related to the chiral feature of surface states. While the other range functions are affected directly by the ferromagnet. Note, moreover, that both $F_1$ and $F_2$ ($F_4$) depend(s) only on the in-plane (out-of-plane) coupling $J_x$ ($J_z$) due to the collinear characteristic of the corresponding interactions. But $F_3$ depends on both $J_x$ and $J_z$ resulting from the non-collinear feature of in-plane Dzyaloshinsky–Moriya interaction. Furthermore, at zero spin-imbalance, i.e., $M = 0$ and $\Gamma _z = 0$, we have $F_1=F_4$ providing isotropic Heisenberg interaction. The integrals of Eqs. (13)–(16) cannot be performed to obtain exact analytical range functions, so, in the following, we first evaluate them numerically and then we use some approximations to get analytical expressions for the range functions in two extreme limits of the model.

**Figure 5:**
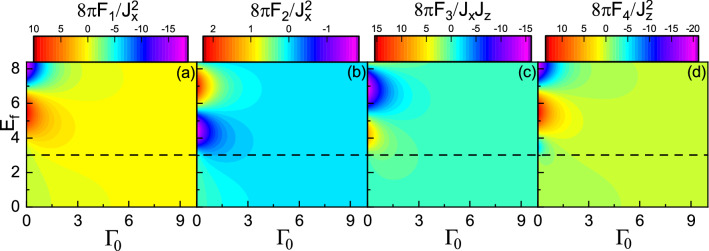
(Color online) **(a)** $F_1$, **(b)** $F_2$, **(c)** $F_3$, and **(d)** $F_4$ as functions of $\Gamma _0$ and $E_f$ with $M=3$, $R=0.5$, and $\Gamma _z=0$. The horizontal dashed line indicates $E_f=M$.

## Numerical results

Given the interest to study indirect exchange interaction in topological insulators, for the sake of completeness, we will present the results in the absence of non-Hermitian terms which might have been derived previously in literature^45,52,53,57^. Without loss of generality, we assume $E_f >0$, set a lattice constant $a=1$ as the length unit, and take $v_f/a = 1$ as the energy unit in the numerical evaluation of the range functions.

### Zero charge and spin transfer case, $\Gamma _0=0$ and $\Gamma _z=0$

Figure 3 shows $F_1$, $F_2$, $F_3$, and $F_4$, as functions of the Fermi energy $E_f$ and impurity distance *R* with $M=3$, in the absence of non-Hermitian terms. The horizontal dash line indicates the boundary between two regimes $E_f > M$ and $E_f < M$, i.e., over gap and subgap regimes. As usual, for $E_f > M$, the range functions oscillate in terms of both $E_f$ and *R*^45,52,57^. With the increase of Fermi energy, the amplitude of spatial oscillations increases, but the period of oscillations decreases, originating from the increase of the Fermi surface^45,52^. On the other hand, for $E_f < M$, the range functions remain unchanged versus $E_f$, whereas the spatial dependence of them tends to zero exponentially^45,53,57^, due to the absence of Fermi surface.

**Figure 6:**
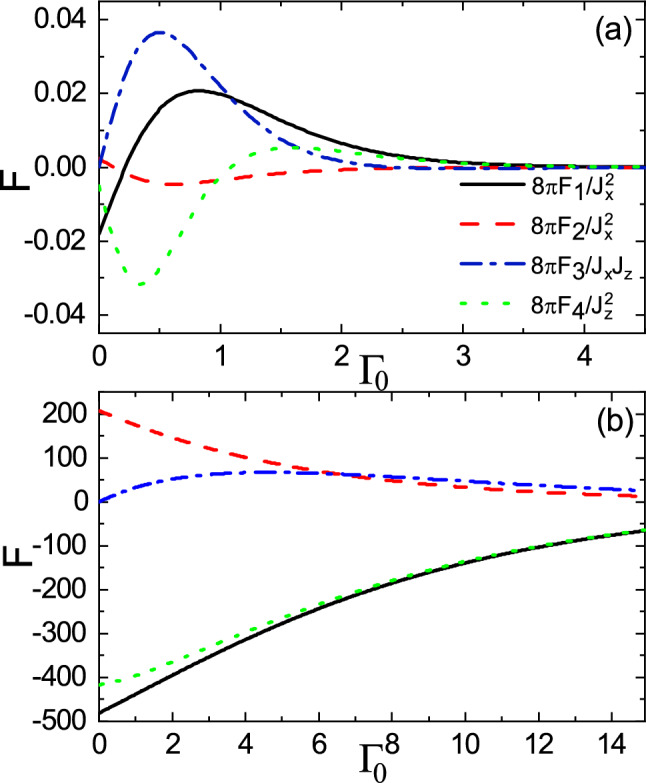
(Color online) Dependence of the range functions on $\Gamma _0$ for **(a)** $R=1$ and **(b)** $R=0.1$ cases in the subgap regime with $E_f=2.5$, $M=3$, and $\Gamma _z=0$.

**Figure 7:**
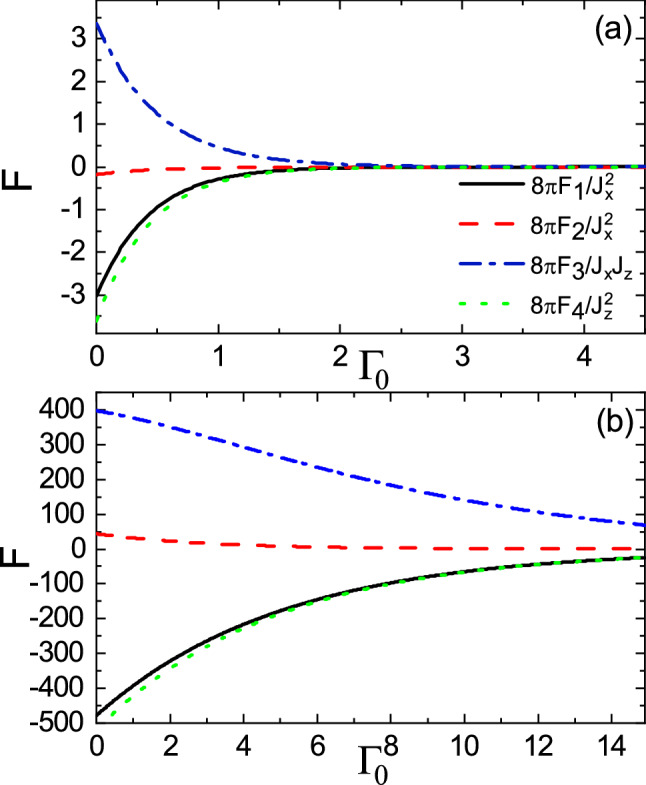
(Color online) Dependence of the range functions on $\Gamma _0$ for **(a)** $R=1$ and **(b)** $R=0.1$ cases in the over gap regime with $E_f=8$, $M=3$, and $\Gamma _z=0$.

In Fig. 4, the *M* and *R* dependence of the range functions are depicted with $E_f=3$. In the over gap regime, with increasing *M*, the amplitude of spatial oscillations decreases but the period of oscillations increases. Also, interestingly, with the further increase of M, near the band edge, the amplitude of range function begins to oscillate as a function of *M*^57^. In the subgap regime, as already mentioned, there is no free carriers. However, the indirect exchange interaction can be mediated through virtual interband transitions of electrons which is known as Bloembergen–Rowland mechanism^58–60^. Note also that from both Figs. 3 and 4, one can see that there is a relatively sharp boundary between the two regimes^45,52,53,57^.

### Non zero charge transfer case, $\Gamma _0\ne 0$ and $\Gamma _z=0$

Now, let us include the charge transfer between the ferromagnet and the topological insulator, i.e., $\Gamma _z=0$. In Fig. 5, the range functions in terms of $E_f$ and $\Gamma _0$ with intermediate distance are shown. For the region $E_f > M$, the oscillations become damped because of escaping the host free carriers weakening indirect exchange interaction. In the case of $E_f < M$, for small $\Gamma _0$, the behavior of the range functions is the same as before. But, interestingly, as $\Gamma _0$ increases, a part of the oscillatory behavior of the over gap regime crosses the boundary (indicated by dashed line) disturbing that of the subgap regime. As a result, the sharp boundary between the two regimes becomes spoiled for large enough $\Gamma _0$. Such behavior can be interpreted as follows. When the imaginary energy states reside deep inside the bandgap, they are out of the reach to play a significant role. But, as $\Gamma _0$ increases, the corresponding imaginary energy states shift towards the band edge. The available imaginary energy states near the bandgap edge promote virtual transitions of electrons between the imaginary energy states and the band edge. This would couple the propagating modes near the band edge to the evanescent ones near the bandgap edge enhancing indirect exchange interaction mediated by Bloembergen–Rowland mechanism at such energies, i.e., $E_f\approx M$. This feature can be seen mathematically in the next section.

For illustrative purposes, in Fig. 6, we have plotted $F_1$, $F_2$, $F_3$, and $F_4$ versus $\Gamma _0$ for the subgap regime with relatively long-range (Fig. 6a) and short-range (Fig. 6b) distances. One can clearly observe that all the range functions behave non-monotonically in the long-range case, as shown in Fig. 6a. We should note that we also examined larger R and the same behavior was found. Furthermore, as plotted in Fig. 6b, in the short-range case, except for the Dzyaloshinsky–Moriya interaction, which remains non-monotonic, the other interactions versus $\Gamma _0$ vanish monotonically. Moreover, in both plots for large $\Gamma _0$, the escape of free carriers is more dominated than the transition from the valence band to the conduction band. So, the range functions tend to vanish. It has been well-studied that in the subgap regime, even for out-of-plane spin-polarized helical spectra, the Dzyaloshinsky–Moriya interaction is negligible^57^ due to vanishingly small in-plane spin-polarized states at the edge of band. But, in the present case, interestingly, the finite values of $\Gamma _0$ promote the Dzyaloshinsky–Moriya interaction.

On the other hand, the dependence of range functions on $\Gamma _0$ in the over gap regime is depicted in Fig. 7. The long-range and short-range behavior are shown in Fig. 7a,b, respectively. As expected, both figures show that the magnitudes of range functions decrease as $\Gamma _0$ increases and the non-trivial feature related to the non-hermiticity vanishes. This is because the energy levels of traveling mode and evanescent mode are not of the same order of magnitude, preventing the coupling between the two modes. However, the decay rate of long-range behavior is larger than that of the short-range one. This can be attributed to the considerable possibility of escaping of carriers when the distance between the magnetic impurities becomes large enough.

**Figure 8:**
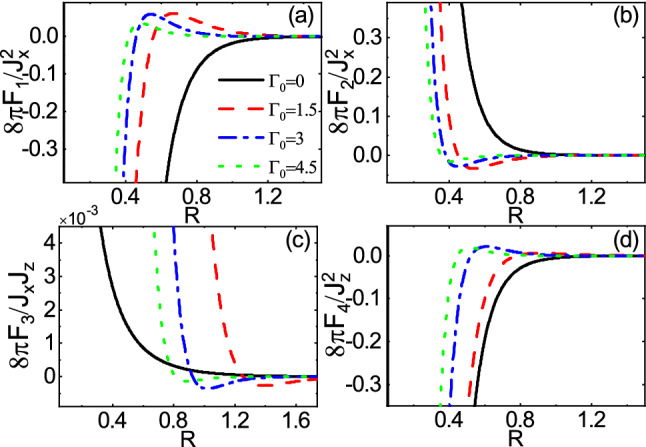
(Color online) R dependence of the range functions **(a)** $F_1$, **(b)** $F_2$, **(c)** $F_3$, and **(d)** $F_4$ for different values of $\Gamma _0$ and $\Gamma _z=0$ in the subgap regime $E_f=2.5$ and $M=3$.

**Figure 9:**
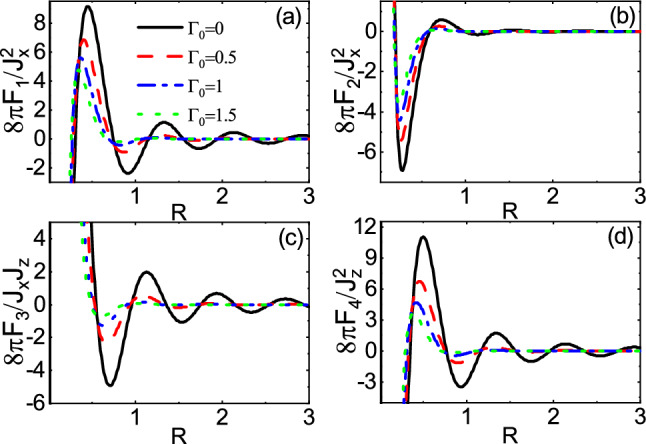
(Color online) R dependence of the range functions **(a)** $F_1$, **(b)** $F_2$, **(c)** $F_3$, and **(d)** $F_4$ for different values of $\Gamma _0$ and $\Gamma _z=0$ in the over gap regime $E_f=5$ and $M=3$.

The spatial dependence of range functions with different values of $\Gamma _0$ is shown in Figs. 8 and 9 for the subgap and the over gap regimes, respectively. In the subgap regime, close to the bandgap edge ($E_f=2.5$ and $M=3$), the range functions fall off deviating from the exponential decay so that the sign change can take place, in contrast to the Hermitian ones^45,57,59,60^. The rate of decay depends on the $\Gamma _0$. As a consequence, unlike the Hermitian indirect exchange interaction where the spin orientation could be either ferromagnetic or aniferromagnetic ordering in the subgap regime, independent of R, in the non-Hermitian case, it is possible to change the spin orientation for one time as R increases. However, in such regime, away from the bandgap edge, the range functions decay exponentially, (not shown) similar to the previous studies^45,57,59,60^. In the over gap regime ($E_f=5$ and $M=3$), the range functions exhibit a damped oscillatory behavior. As already mentioned, the amplitudes of oscillations decrease with increasing $\Gamma _0$. Also, the period of oscillations decreases steaming from increasing the real part of Fermi momentum due to $\Gamma _0$.

### Non zero spin transfer case, $\Gamma _0=0$ and $\Gamma _z\ne 0$

**Figure 10:**
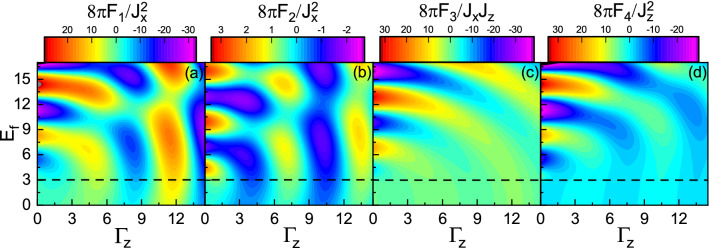
(Color online) **(a)** $F_1$, **(b)** $F_2$, **(c)** $F_3$, and **(d)** $F_4$ versus $\Gamma _z$ and $E_f$ with $M=3$, $R=0.5$, and $\Gamma _0=0$. The horizontal dashed line indicates $E_f=M$.

Now, we consider the spin transfer between the ferromagnet and the topological insulator, i.e., $\Gamma _0=0$. In Fig. 10, the dependence of the range functions on $\Gamma _z$ and on $E_f$ is shown. Surprisingly, in addition to traveling states close the band edge, the higher energy spin states shift toward the evanescent states as $\Gamma _z$ increases at the intermediate distances. Because of spin-split imaginary states above and below the Dirac point (See Fig. 2c), virtual electron-hole excitations would be promoted resulting in a strong coupling between the two types of modes. Such strong coupling causes the periodicity in terms of $\Gamma _z$ to be inherited from the periodicity of Fermi energy.

**Figure 11:**
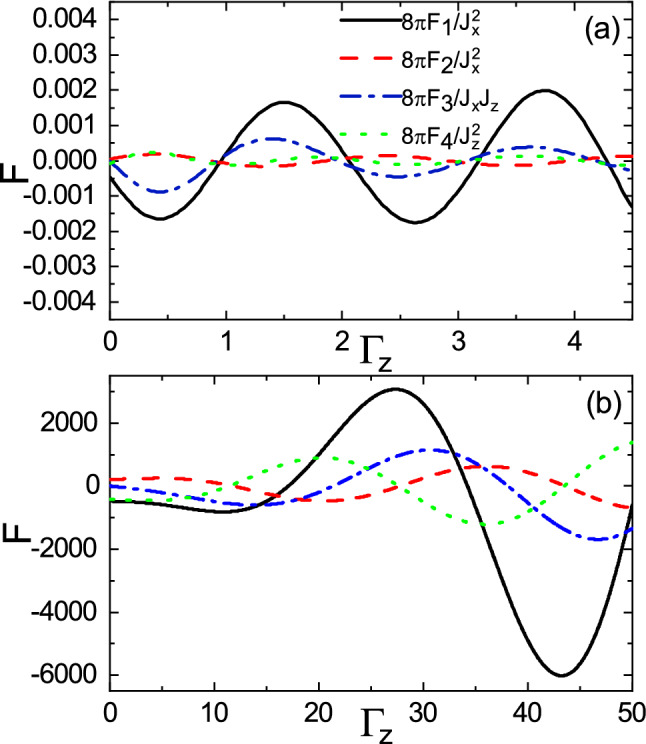
(Color online) Dependence of the range functions on $\Gamma _z$ for **(a)** $R=1.5$ and **(b)** $R=0.1$ in the subgap regime with $E_f=2$, $M=3$, and $\Gamma _0=0$.

In the subgap regime, the long-range and short-range behaviors of the range functions as a function of $\Gamma _z$ are shown in Fig. 11a,b, respectively. The range functions exhibit oscillatory behavior with the same period in the long-range case (see Fig. 11a). For the short-range case, while the range functions are almost constant when $\Gamma _z$ is small (see Fig. 11b), they begin to oscillate versus the spin decay rate as $\Gamma _z$ exceeds some certain value.

**Figure 12:**
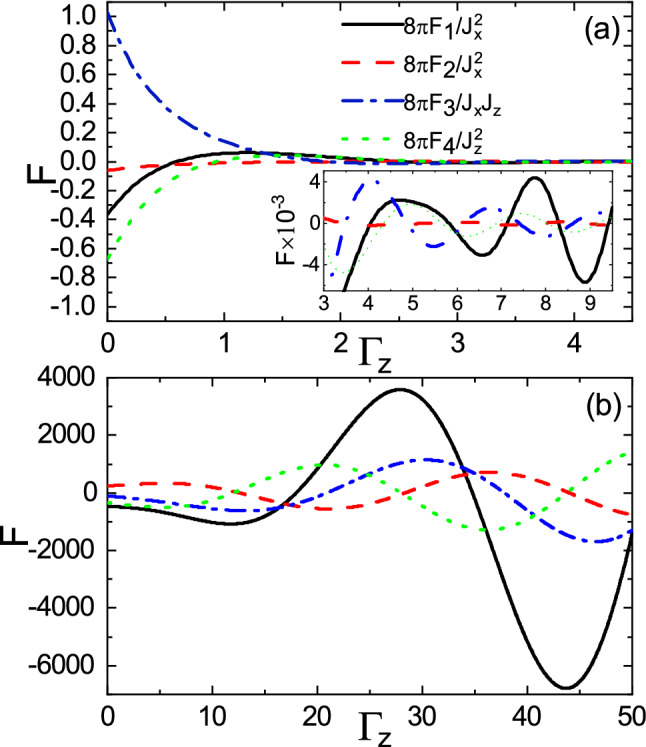
(Color online) Dependence of the range functions on $\Gamma _z$ for **(a)** $R=1$ and **(b)** $R=0.1$ cases in the over gap regime with $E_f=5$, $M=3$, and $\Gamma _0=0$.

Furthermore, the long-range and short-range behaviors of the range functions versus $\Gamma _z$ in the over gap regime are shown in Fig. 12a,b, respectively. In the long-range case, the amplitudes of range functions decease (see the main panel of Fig. 12a), and then with further increase of $\Gamma _z$, the range functions reveal oscillations with small amplitudes (see the inset of Fig. 12a). Also, as shown in Fig. 12b, the short distance behavior of range functions in the over gap regime is similar to that of range functions in the subgap regime (see Fig. 11b). In this case, the amplitudes of range functions are slightly enhanced compared with those in the subgap case.

**Figure 13:**
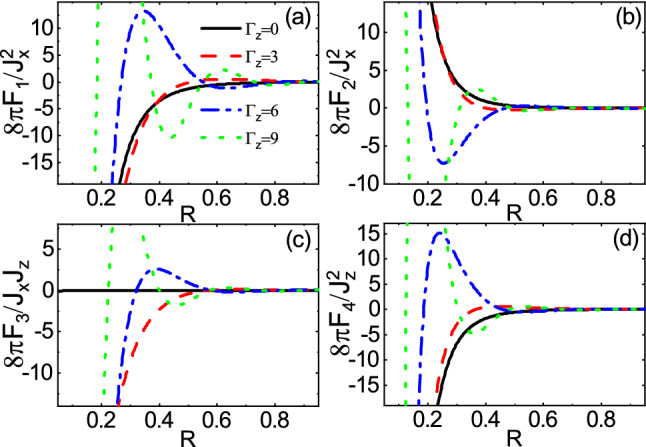
(Color online) R dependence of the range functions **(a)** $F_1$, **(b)** $F_2$, **(c)** $F_3$, and **(d)** $F_4$ for different values of $\Gamma _z$ and $\Gamma _0=0$ in the subgap regime $E_f=2$ and $M=3$.

**Figure 14:**
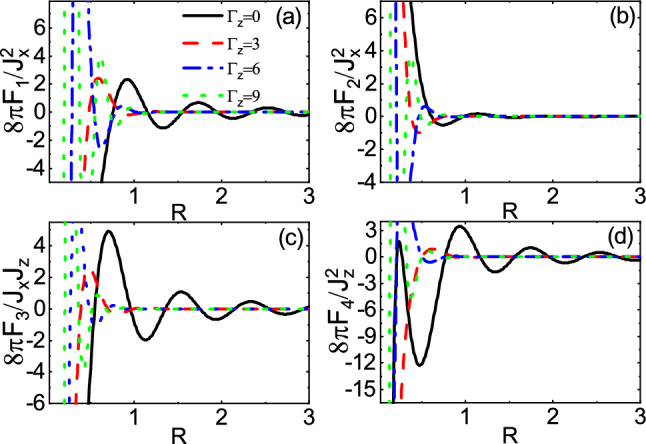
(Color online) R dependence of the range functions **(a)** $F_1$, **(b)** $F_2$, **(c)** $F_3$, and **(d)** $F_4$ for different values of $\Gamma _z$ and $\Gamma _0=0$ in the over gap regime $E_f=5$ and $M=3$.

Owing to coupling of the evanescent modes with the traveling modes, in the subgap regime, the range functions decay and at the same time, interestingly, oscillate in space as shown in Fig. 13. As $\Gamma _z$ increases, the amplitudes of oscillations increase whereas the periods of oscillations decrease. As a consequence, the non-Hermitian term $\Gamma _z$ can enhanced the interactions and can provide several possibilities for ferromagnetic-antiferromagnetic transition in space which are in sharp contrast to the Hermitian indirect exchange interaction. In the over gap regime, as shown in Fig. 14, similar to Fig. 9, the period of oscillations decreases, as $\Gamma _z$ increases. In contrast, the range functions strongly oscillate at small *R* whereas the spatial oscillations become more damped than those of $\Gamma _z=0$ case at large *R*. As a result, the long-range interactions in the over gap regime turn into the short-range ones due to $\Gamma _z$.

### Mixed charge and spin transfer case, $\Gamma _0\ne 0$ and $\Gamma _z\ne 0$

**Figure 15:**
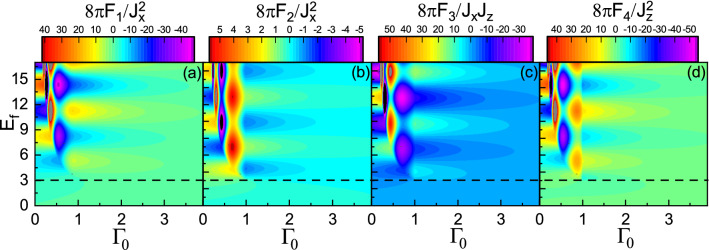
(Color online) **(a)** $F_1$, **(b)** $F_2$, **(c)** $F_3$, and **(d)** $F_4$ versus $\Gamma _0$ and $E_f$ with $\Gamma _z=1$, $M=3$, and $R=0.5$. The horizontal dashed line indicates $E_f=M$.

**Figure 16:**
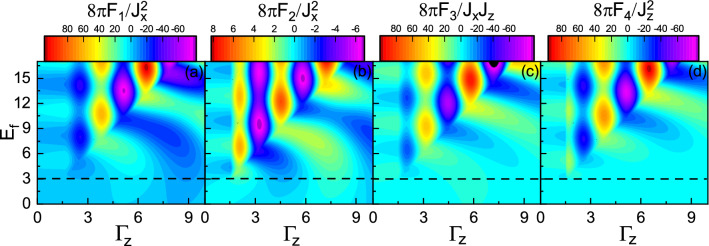
(Color online) **(a)** $F_1$, **(b)** $F_2$, **(c)** $F_3$, and **(d)** $F_4$ versus $\Gamma _z$ and $E_f$ with $\Gamma _0=1.5$, $M=3$ and $R=0.5$. The horizontal dashed line indicates $E_f=M$.

Now, we include both $\Gamma _0$ and $\Gamma _z$ in order to investigate their mutual effects. In Figs. 15 and 16, the range functions are plotted in the plane ($\Gamma _0$, $E_f$) and ($\Gamma _z$, $E_f$) with $\Gamma _z=1$ and $\Gamma _0=1.5$, respectively. Comparing these figures with Figs. 5 and 10, one finds that the patterns of range functions are disturbed so that for given values of $E_f$ and $\Gamma _0$ ($\Gamma _z$) there are very sharp sign changes at certain values of $\Gamma _z$ ($\Gamma _0$) in the over gap regime resulting in the parallel-antiparallel transition of magnetic spin alignment. As one can see from Fig. 15, these sign changes are restricted below certain values of $\Gamma _0$. But Fig. 16 shows that the sign changes can be happened at large $\Gamma _z$ when $E_f$ increases. In addition, the abrupt sign changes of the over gap regime is extended to the subgap regime with smoother behavior. As a result, unlike the cases where only one of the decay rates is present and the main change takes place in the subgap regime, the presence of both types of the decay rates would substantially affect the over gap regime. This arises from the asymmetric spin-splitting of imaginary states (see Fig. 2d).

## Analytical results

In this section, we obtain approximate analytic expressions for the indirect exchange coupling and exhibit their dominant dependence on the parameters. To do so, we consider the two extreme limits of the model, namely, short distance and long distance behaviors. Since most of the features, discussed above, are pronounced close to the bandgap edge, in the following, we restrict ourself to the case $E_f\approx M$ to obtain relatively short expressions.

### Non zero charge transfer case, $\Gamma _0\ne 0$ and $\Gamma _z=0$

In the long-range limit, $x\rightarrow \infty$, inserting the asymptotic expression of Hankel functions^62^ into Eqs. (13)–(16). For $M\gg \Gamma _0$, we can use the small variable expansions of the integrals and expand the integrands up to second order. After performing the integrals, the dominant terms can be reduced to

17 $$\begin{aligned} F_1\!\simeq & {} \! -\frac{J^2_x}{2\pi ^{2}v_F^2R^2}\Gamma _0e^{-\frac{2\alpha R}{v_F}} \cos \left( \frac{2\alpha R}{v_F}\right) , \end{aligned}$$

18 $$\begin{aligned} F_2\!\simeq & {} \! \frac{J^2_x}{4\pi ^{2}v_F^2R^2} \frac{\Gamma ^2_0}{M}e^{-\frac{2 \alpha R}{v_F}} \sin \left( \frac{2\alpha R}{v_F}\right) ,\end{aligned}$$

19 $$\begin{aligned} F_3\!\simeq & {} \! \frac{J_xJ_z}{4\pi ^{2}v_F^2 R^2}\alpha e^{-\frac{2\alpha R}{v_F}}\left[ \cos \left( \frac{2\alpha R}{v_F}\right) -\sin \left( \frac{2\alpha R}{v_F}\right) \right] ,\end{aligned}$$

20 $$\begin{aligned} F_4\!\simeq & {} \! -\frac{J^2_z}{4\pi ^{2}v_F^2R^2}Me^{-\frac{2\alpha R}{v_f}} \sin \left( \frac{2\alpha R}{v_F}\right) , \end{aligned}$$

where $\alpha =\sqrt{M\Gamma _0}$. In the equations above, there is an exponential factor depending on the R, $\Gamma _0$, and *M* implying that, in addition to the distance and gap, the charge decay rate causes the exponential decaying. Also, $F_1$, $F_2$, and $F_3$ are proportional to $\Gamma _0$, $\Gamma _0^2$, and $\sqrt{M\Gamma _0}$, respectively, causing the non-monotonic behavior of the range function versus $\Gamma _0$. The range function of Ising interaction, $F_4$, is proportional to *M* as it must be. The presence of cosine and sine functions is responsible for the sign change of the range functions. Note, interestingly, that the range functions fall off exponentially, as already discussed, but at the same time, show the power-law decay $R^2$, in contrast to the previous studies^45,53,57^. Note that the exponential and harmonic functions originate from the wavefunction of evanescent and traveling modes, respectively. The product of the two functions, depending on the decay rate, reveals the coupling of the evanescent and traveling modes, as already mentioned above.

On the other hand, in the short distance case, for $\Gamma _0\gg M$, we can expand the integrands of Eqs. (13)–(16) as a power series of M. After integration, we obtain the dominant terms

21 $$\begin{aligned} F_1\!\simeq & {} \!-\frac{J^2_x}{8\pi ^{5/2}v_FR^3}\! \left[ \!{\mathscr {M}}_1(x)\!+\!{\mathscr {M}}_2(x)\!+\!\frac{M^2R^2{\mathscr {M}}_3(x)}{v_F^2}\!\right] ,\end{aligned}$$

22 $$\begin{aligned} F_2\!\simeq & {} \!-\frac{J^2_x}{8\pi ^{5/2}v_FR^3}\! \left[ \!{\mathscr {M}}_1(x)\!-\!{\mathscr {M}}_2(x)\!+\!\frac{M^2R^2{\mathscr {M}}_3(x)}{v_F^2}\!\right] ,\end{aligned}$$

23 $$\begin{aligned} F_3\!\simeq & {} \! \frac{J_xJ_z}{\pi ^3v_F^3R} M\Gamma _0 K^2_1(x),\end{aligned}$$

24 $$\begin{aligned} F_4\!\simeq & {} \!-\frac{J^2_z}{8\pi ^{5/2}v_FR^3}\! \left[ \!{\mathscr {M}}_1(x)\!+\!{\mathscr {M}}_2(x)\!-\!\frac{M^2R^2{\mathscr {M}}_3(x)}{v_F^2}\!\right] . \end{aligned}$$

where $$x=\Gamma _0\frac{R}{v_F}$$ and

25 $$\begin{aligned} {\mathscr {M}}_1(x)= \,& {} G^{4,0}_{2,4}\left( x^2\big \vert \begin{array}{c} 1,2 \\ 0,\frac{3}{2},\frac{3}{2},\frac{3}{2} \end{array} \right) , \end{aligned}$$

26 $$\begin{aligned} {\mathscr {M}}_2(x)=\, & {} G^{4,0}_{2,4}\left( x^2\big \vert \begin{array}{c} 1,2 \\ 0,\frac{1}{2},\frac{3}{2},\frac{5}{2} \end{array} \right) , \end{aligned}$$

27 $$\begin{aligned} {\mathscr {M}}_3(x)= \,& {} G^{4,0}_{2,4}\left( x^2\big \vert \begin{array}{c} 1,1 \\ 0,\frac{1}{2},\frac{1}{2},\frac{1}{2} \end{array} \right) , \end{aligned}$$

are the Meijer G-functions and $K_1(x)$ is the modified Bessel function of the first kind. Using the asymptotic expressions for the ${\mathscr {M}}_1(x)$, ${\mathscr {M}}_2(x)$, ${\mathscr {M}}_3(x)$, and $K_1(x)$ in the limit $x\rightarrow 0$^61,62^, one finds

28 $$\begin{aligned} F_1\!\simeq \,& {} \!-\frac{J^2_x}{2\pi ^{3}v_FR^3}\! \left[ \frac{\pi ^2}{8}-\Gamma _0\frac{R}{v_F}+\frac{\pi ^2}{4} M^2\frac{R^2}{v^2_F} \right] ,\end{aligned}$$

29 $$\begin{aligned} F_2\!\simeq \,& {} \!\frac{J^2_x}{2\pi ^{3}v_FR^3}\! \left[ \frac{\pi ^2}{16}-\Gamma _0\frac{R}{v_F}-\frac{\pi ^2}{4}M^2\frac{R^2}{v^2_F}\right] ,\end{aligned}$$

30 $$\begin{aligned} F_3\!\simeq \,& {} \! \frac{J_xJ_z}{\pi ^3v_FR^3} \frac{M}{\Gamma _0}\! \left[ \!1+\Gamma _0^2\frac{R^2}{v^2_F}\left(Ln\left( \frac{e^{\gamma }\Gamma _0}{2}\frac{ R}{v_F}\right)-\frac{1}{2}\right) \!\right] ,\end{aligned}$$

31 $$\begin{aligned} F_4\!\simeq \,& {} \!-\frac{J^2_z}{2\pi ^{3}v_FR^3}\! \left[ \frac{\pi ^2}{8}-\Gamma _0\frac{R}{v_F}-\frac{\pi ^2}{4}M^2\frac{R^2}{v^2_F} \right] . \end{aligned}$$

Here, $\gamma$ is the Euler–Mascheroni constant. One can see that the dominant terms decay as a power law of $R^{-3}$. Note that the presence of parameters *M* and $\Gamma _0$ only modifies the $F_1$, $F_2$,and $F_4$. But non zero values of $F_3$ is due to these parameters (see also Eq. (23)).

### Non zero spin transfer case, $\Gamma _0=0$ and $\Gamma _z\ne 0$

In the long-range limit, $x\rightarrow \infty$, similar to the previous subsection for $M\gg \Gamma _z$, one gets

32 $$\begin{aligned} F_1\!\simeq \,& {} \! \frac{J^2_x}{2\pi ^{2}v_F^2R^2} \Gamma _ze^{-\frac{2\alpha ^{\prime } R}{v_F}} \cos \left( \frac{2\alpha ^{\prime } R}{v_F}\right) ,\end{aligned}$$

33 $$\begin{aligned} F_2\!\simeq\, & {} \! -\frac{J^2_x}{16\pi ^{2}v_F^2R^2} Me^{-\frac{2 M R}{v_f}} \sin \left( \frac{2\alpha ^{\prime } R}{v_F}\right) ,\end{aligned}$$

34 $$\begin{aligned} F_3\!\simeq \,& {} \! -\frac{J_xJ_z}{4\pi ^{2}v_F^2R^2} \alpha ^{\prime } e^{-\frac{2\alpha ^{\prime } R}{v_F}}\left[ \cos \left( \frac{2\alpha ^{\prime } R}{v_F}\right) -\sin \left( \frac{2\alpha ^{\prime } R}{v_F}\right) \right] ,\end{aligned}$$

35 $$\begin{aligned} F_4\!\simeq \,& {} \! \frac{J^2_z}{4\pi ^{2}v_F^2R^2} M e^{-\frac{2\alpha ^{\prime } R}{v_F}} \sin \left( \frac{2\alpha ^{\prime } R}{v_F}\right) , \end{aligned}$$

where $\alpha ^{\prime }=\sqrt{M\Gamma _z}$. Similarly, one can see that the exponential decay is accompanied with the oscillatory behavior. Following similar steps to those in the previous subsection, in the short distance case for $M \gg \Gamma _z$, Eqs. (13)–(16) can be obtained approximately in terms of the Meijer G-functions as

36 $$\begin{aligned} F_1\!\simeq \,& {} \!-\frac{J^2_x}{8\pi ^3v_FR^3}\! Re\!\!\left[ \!\frac{y^2{\mathscr {M}}^{\prime }_1(y)}{2}\!+\!y^4{\mathscr {M}}^{\prime }_2(y)\!+\!y^4{\mathscr {M}}^{\prime }_3(y)\!\right] ,\end{aligned}$$

37 $$\begin{aligned} F_2\!\simeq\, & {} \!- \frac{J^2_x}{8\pi ^3v_FR^3}\! Re\!\!\left[ \!\frac{y^2{\mathscr {M}}^{\prime }_1(y)}{2}\!-\!y^4{\mathscr {M}}^{\prime }_2(y)\!+\!y^4{\mathscr {M}}^{\prime }_3(y)\!\right] ,\end{aligned}$$

38 $$\begin{aligned} F_3\!\,= \,& {} \!- \frac{J_xJ_z}{\pi ^3v_F^3R} M\Gamma _z Re \left[ K^2_1(\frac{\sqrt{2i M\Gamma _z}R}{v_F})\right] ,\end{aligned}$$

39 $$\begin{aligned} F_4\!\simeq\, & {} \!-\frac{J^2_z}{8\pi ^3v_FR^3}\! Re\!\!\left[ \!\frac{y^2{\mathscr {M}}^{\prime }_1(y)}{2}\!+\!y^4{\mathscr {M}}^{\prime }_2(y)\!-\!y^4{\mathscr {M}}^{\prime }_3(y)\!\right] . \end{aligned}$$

where $$y=(M+i\Gamma _z)\frac{R}{v_F}$$ and

40 $$\begin{aligned} {\mathscr {M}}^{\prime }_1(y)=\, & {} G^{4,1}_{2,4}\left( -y^2\big \vert \begin{array}{c} \frac{1}{2},1 \\ -1,\frac{1}{2},\frac{1}{2},\frac{1}{2} \end{array} \right) , \end{aligned}$$

41 $$\begin{aligned} {\mathscr {M}}^{\prime }_2(y)=\, & {} G^{4,1}_{2,4}\left( -y^2\big \vert \begin{array}{c} -\frac{3}{2},0 \\ -2,-\frac{3}{2},-\frac{1}{2},\frac{1}{2} \end{array} \right) , \end{aligned}$$

42 $$\begin{aligned} {\mathscr {M}}^{\prime }_3(y)= \,& {} G^{4,1}_{2,4}\left( -y^2\big \vert \begin{array}{c} -\frac{1}{2},0 \\ -1,-\frac{1}{2},-\frac{1}{2},-\frac{1}{2} \end{array} \right) . \end{aligned}$$

In the limit $y\rightarrow 0$, the asymptotic expressions of ${\mathscr {M}}^{\prime }_1(y)$, ${\mathscr {M}}^{\prime }_2(y)$, ${\mathscr {M}}^{\prime }_3(y)$, and $K_1(x)$ can be used^61,62^ yielding,

43 $$\begin{aligned} F_1\!\simeq \,& {} \! -\frac{J^2_x}{2\pi ^3v_FR^3}\!\left[ \frac{\pi ^2}{8}-\Gamma _z\frac{R}{v_F}-\frac{\pi ^2}{2}M^2\frac{R^2}{v_F^2}\!\right] ,\end{aligned}$$

44 $$\begin{aligned} F_2\!\simeq \,& {} \! \frac{J^2_x}{2\pi ^3v_FR^3}\!\left[ \frac{\pi ^2}{16}-\Gamma _z\frac{R}{v_F}+\frac{\pi ^2}{4}(M^2-2M\Gamma _z)\frac{R^2}{v_F^2}\!\right] ,\end{aligned}$$

45 $$\begin{aligned} F_3\!\simeq \,& {} \!- \frac{J_xJ_z}{4\pi ^3v_FR^3}\frac{\Gamma _z}{M}\! \left[ 1+4M^2\frac{R^2}{v^2_F}\left( Ln\left( \frac{e^{\gamma }\alpha ^{\prime }}{\sqrt{2}}\frac{R}{v_F}\right) -\frac{1}{2}\right) \!\right] ,\end{aligned}$$

46 $$\begin{aligned} F_4\!\simeq \,& {} \! -\frac{J^2_z}{2\pi ^3v_FR^3}\left[ \frac{\pi ^2}{8}-\Gamma _z\frac{R}{v_F}\right] . \end{aligned}$$

One can see that, in this case, the gap and the spin decay rate modify the range functions except for $F_3$. Again, at such distances, the range functions fall off as $R^{-3}$.

## Summary

In conclusion, we have explored the effects of non-Hermiticity on the indirect exchange interaction mediated by the surface states of the topological insulator that is in contact with the ferromagnetic metal. The non-Hermiticity arises as a result of coupling between the topological insulator and the ferromagnetic metal, inducing loss and gain of quasi-particles. It turns out that the charge (spin) decay rate can weakly (strongly) couple the traveling modes to the evanescent modes.

In the subgap regime, when the Fermi energy lies close to the bandgap edge, all the range functions change non-monotonically with the distance between magnetic impurities as well as with the charge decay rate, and then tend to zero as the charge decay rate increases. In the over gap regime, the oscillatory behavior dependence of the range functions on distance is damped by the charge decay rate at both long- and short-distance limits.

Most interestingly, in the presence of spin decay rate, in the subgap regime, the interaction terms oscillate as a function of distance with exponential envelope. While in the over gap regime, the spin decay rate decreases (increases) the amplitude of oscillations at long (short) distances.

Usually, in the Hermitian indirect exchange interaction, for given parameters such as distance and Fermi energy, it is difficult to quench all types of interaction simultaneously. But for the non-Hermitian indirect exchange interaction, as already discussed, at the large value of charge decay rate, it is possible to turn off all the types of interaction independent of the other parameters. Remarkably, the spin decay rate can enhance the indirect exchange interaction and change the spin impurity alignment. These features would provide more control to manipulate spin impurity interaction which are key requirements in potential applications, for instance, quantum spin memory as well as quantum computations.
